# Long Non-Coding RNA TMPO-AS1 Promotes GLUT1-Mediated Glycolysis and Paclitaxel Resistance in Endometrial Cancer Cells by Interacting With miR-140 and miR-143

**DOI:** 10.3389/fonc.2022.912935

**Published:** 2022-05-27

**Authors:** Peixin Dong, Feng Wang, Mohammad Taheri, Ying Xiong, Kei Ihira, Noriko Kobayashi, Yosuke Konno, Junming Yue, Hidemichi Watari

**Affiliations:** ^1^ Department of Obstetrics and Gynecology, Hokkaido University School of Medicine, Hokkaido University, Sapporo, Japan; ^2^ Department of Laboratory Medicine, Affiliated Hospital of Nantong University, Jiangsu, China; ^3^ Skull Base Research Center, Loghman Hakim Hospital, Shahid Beheshti University of Medical Sciences, Tehran, Iran; ^4^ Institute of Human Genetics, Jena University Hospital, Jena, Germany; ^5^ Department of Gynecology, State Key Laboratory of Oncology in South China, Sun Yat-sen University Cancer Center, Guangzhou, China; ^6^ Department of Pathology and Laboratory Medicine, University of Tennessee Health Science Center, Memphis, TN, United States; ^7^ Center for Cancer Research, University of Tennessee Health Science Center, Memphis, TN, United States

**Keywords:** GLUT1, miR-140, miR-143, long non-coding RNA, TMPO-AS1, paclitaxel resistance, glycolysis, endometrial cancer

## Abstract

Increased glycolysis in tumor cells is frequently associated with drug resistance. Overexpression of glucose transporter-1 (GLUT1) promotes the Warburg effect and mediates chemoresistance in various cancers. Aberrant GLUT1 expression is considered as an essential early step in the development of endometrial cancer (EC). However, its role in EC glycolysis and chemoresistance and the upstream mechanisms underlying GLUT1 overexpression, remain undefined. Here, we demonstrated that GLUT1 was highly expressed in EC tissues and cell lines and that high GLUT1 expression was associated with poor prognosis in EC patients. Both gain-of-function and loss-of-function studies showed that GLUT1 increased EC cell proliferation, invasion, and glycolysis, while also making them resistant to paclitaxel. The long non-coding RNA TMPO-AS1 was found to be overexpressed in EC tissues and to be negatively associated with EC patient outcomes. RNA-immunoprecipitation and luciferase reporter assays confirmed that TMPO-AS1 elevated GLUT1 expression by directly binding to two critical tumor suppressor microRNAs (miR-140 and miR-143). Downregulation of TMPO-AS1 remarkably reduced EC cell proliferation, invasion, glycolysis, and paclitaxel resistance in EC cells. This study established that dysregulation of the TMPO-AS1-miR-140/miR-143 axis contributes to glycolysis and drug resistance in EC cells by up-regulating GLUT1 expression. Thus, inhibiting TMPO-AS1 and GLUT1 may prove beneficial in overcoming glycolysis-induced paclitaxel resistance in patients with EC.

## Introduction

A hallmark of cancer is the remodeling of glucose metabolism (1). Even in the presence of abundant oxygen, cancer cells exhibit enhanced glycolysis to generate energy and supply intermediates for biosynthetic processes (1). Since cancer cells rely on increased glucose usage compared with normal healthy cells, inhibition of glucose metabolism represents a promising therapeutic option in combating cancer (2).

The glucose transporter (GLUT) proteins are a class of membrane proteins that aid in glucose transport across the plasma membrane (1). Cancer cells frequently overexpress GLUTs, particularly GLUT1, which significantly increases glucose import into the cytoplasm (3). Overexpression of GLUT1 has been observed in many types of cancers, and high GLUT1 expression is associated with unfavorable overall survival in human cancers (4, 5). GLUT1 has been shown to promote cancer cell proliferation, migration, invasion, and metastasis (6, 7). Accumulated reports indicate that aerobic glycolysis is linked to tumor chemoresistance (8, 9). Inhibition of GLUT1 sensitized tumor cells to the anti-cancer effects of chemotherapeutic drugs (10–12), although the mechanisms remain elusive.

GLUT1 expression is usually absent in benign endometrium (13, 14). However, GLUT1 expression was preferentially expressed in atypical hyperplasia and endometrial cancer (EC) specimens (13, 14), indicating a biological role for GLUT1 during the early stages of endometrial tumorigenesis. GLUT1 expression progressively increased from low-grade EC to high-grade EC (15). A recent study showed that treating spheroid cells derived from EC patients with BAY876, a specific GLUT1 inhibitor, could sensitize them to paclitaxel and suppress their glucose uptake (16). The potential roles of GLUT1 in regulating glycolysis and paclitaxel resistance in EC cells, as well as the underlying mechanisms, have not yet been elucidated.

Numerous non-coding RNAs, including microRNAs (miRNAs) and long non-coding RNAs (lncRNAs), have been identified to regulate cancer metabolism and chemoresistance (17). LncRNAs play an important role in regulating gene expression at multiple levels (including epigenetic, transcription, and post-transcriptional regulation) (18). Notably, lncRNAs act as competing endogenous RNAs, antagonizing the interactions of tumor suppressor miRNAs and target mRNAs (18). However, the epigenetic mechanisms underlying GLUT1 overexpression in EC, and the key molecular machinery by which lncRNAs mediate glycolysis and paclitaxel resistance *via* miRNAs, have not been identified.

In this study, we revealed a critical function of GLUT1 that facilitates glycolysis and confers paclitaxel resistance in EC cells. The interactions between lncRNA TMPO-AS1 and miR-140/miR-143 protect GLUT1 from miRNA inhibition, thus promoting glycolysis and paclitaxel resistance. This is the first report suggesting that TMPO-AS1 binds directly to miR-140/miR-143 to induce the expression of GLUT1 in EC cells, implying that GLUT1 is a downstream target of the TMPO-AS1-miR-140/miR-143 pathway. Furthermore, high GLUT1 and TMPO-AS1 expression is correlated with poor overall survival in EC patients. Our findings point to GLUT1 and TMPO-AS1 as potential therapeutic targets for improving the prognosis of EC patients by inhibiting glycolysis and overcoming paclitaxel resistance.

## Materials and Methods

### Bioinformatic Analysis

The Oncomine database (https://www.oncomine.org/resource/login.html) was used to detect *GLUT1* expression in several types of cancer and normal tissues with the thresholds (*P* < 0.05, a 2-fold change, and a gene rank in the top 10%). The GEO datasets (GSE17025 and GSE63678) were combined to identify differentially expressed genes between EC and normal tissues using the ImaGEO database (https://imageo.genyo.es), which annotated the probes with gene identifiers, merged and normalized data. The Human Protein Atlas (HPA) is an open-access database that maps all human proteins in cells and tissue samples (https://www.proteinatlas.org/). Immunohistochemistry (IHC) pictures of EC tissues were retrieved from the HPA. The KM plotter database (http://kmplot.com/analysis/) was used to analyze the effects of *GLUT1* expression on the overall patient survival. The *P*-value was determined using the log-rank method. Genes associated with *GLUT1* were investigated using the LinkedOmics database (http://www.linkedomics.org). Kyoto Encyclopedia of Genes and Genomes (KEGG) and Wikipathway cancer enrichment analysis was carried out by Gene Set Enrichment Analysis (GSEA) in LinkedOmics. To select significantly enriched gene sets, 1000 simulations are conducted, and the false discovery rate (FDR < 0.05) was used. The expression of TMPO-AS1 in TCGA EC and normal tissues was examined using the ENCORI database (http://starbase.sysu.edu.cn) and Wanderer database (http://maplab.imppc.org/wanderer/). TANRIC database (https://www.tanric.org/) was applied to investigate the expression of TMPO-AS1 in TCGA EC patients with different tumor grades and stages.

### Human Cell Lines and Cell Culture

Human EC cell lines (Ishikawa and HHUA) were derived from well-differentiated endometrioid EC. Ishikawa cells were obtained from the American Type Culture Collection (ATCC, Manassas, VA), and HHUA cells were purchased from the RIKEN cell bank (Tsukuba, Japan). An immortalized human endometrial epithelial cell line (EM) was a kind gift from Dr. Satoru Kyo (Shimane University, Japan). These cells were cultured in DMEM/F12 medium (Sigma-Aldrich, St. Louis, MO) that contained 10% fetal bovine serum (FBS, Invitrogen, Carlsbad, CA) and 100 μg/ml Normocin (Invitrogen, San Diego, CA, USA) at 37°C with 5% CO_2_.

### Plasmids and Transfection

To generate stable HHUA cell lines overexpressing GLUT1, the *GLUT1* cDNA expression vector pCMV6-GLUT1 (GLUT1-vec) or control vector was purchased from OriGene (Rockville, MD). Cell transfections were performed using Lipofectamine 3000 reagent (Invitrogen) following the manufacturer’s instructions. The resulting cell lines were expanded after 14 days of selection with G418 (500 μg per ml, Sigma-Aldrich, St. Louis, MO).

*GLUT1* silencing was conducted in the Ishikawa cell line using shRNA targeting GLUT1 (Santa Cruz Biotechnologies, Santa Cruz, CA) or control shRNA (Santa Cruz Biotechnologies) and Lipofectamine 3000 reagent. The transfected cells were expanded after being selected with puromycin (1 μg per ml, Sigma-Aldrich, St. Louis, MO) for 14 days. Western blotting analysis was used to evaluate forced GLUT1 overexpression or knockdown.

The control (NC) mimic, miR-140 mimic, miR-143 mimic, control inhibitor, miR-140 inhibitor, miR-143 inhibitor, control siRNA, and a specific siRNA against TMPO-AS1 were obtained from Invitrogen. Transfection of EC cells was performed using Lipofectamine 3000 reagent (Invitrogen). After transfection, cells were incubated for 48 hours before proceeding with the experiments.

### Western Blotting Analysis

Whole-cell extracts were prepared in M-Per Mammalian Protein Extraction Reagent (Pierce, Rockford, IL), separated by SDS-polyacrylamide gels, and transferred to PVDF membranes (GE Healthcare Life Sciences, Piscataway, NJ). Membranes were incubated overnight with primary antibodies, including anti-GLUT1 (1:1000, Cell Signaling Technology, Beverly, MA) and anti-β-actin (1:5000, Cell Signaling Technology). Protein bands were detected with an ECL detection kit (Amersham Pharmacia Biotech, UK).

### CCK-8 Assay

Cell proliferation was assessed using the Cell Counting Kit-8 (CCK-8) assay (Dojindo, Japan) according to the manufacturer’s instructions. Five thousand cells were seeded per well in a 96-well plate and cultured for 72 hours. For the paclitaxel viability assays, 5000 cells per well were plated in 96-well plates for 24 hours and then treated with DMSO or varying doses of paclitaxel (Cell Signaling Technology). After 24 hours of paclitaxel treatment, cell viability was determined using the CCK-8 assay. The relative survival was calculated compared with cells treated with DMSO.

### Matrigel Invasion Assay

Matrigel invasion assays were performed as previously reported (19). A total of 5 × 10^4^ cells were plated in the upper wells of Matrigel−coated Transwell plates (8 µm pore size, Corning Costar Co., Lowell, CA) in 500 μl of serum-free DMEM/F12 medium containing 100 μg/ml of Normocin. The lower chambers of the plate were filled with 750 µl of DMEM/F12 medium containing 10% FBS and 100 μg/ml of Normocin. The cells were allowed to invade for 24 hours before being stained with 2% crystal violet for 15 minutes. Cells in the upper chamber were removed using a cotton swab, and cells migrating through the membrane were counted.

### Measurement of Glucose Consumption and Lactate Production

Cells were cultured in DMEM/F12 medium in 6-well plates, and the medium was changed after 6 hours. Culture media was collected after incubation for 24 hours. The glucose concentration in the medium was determined using the Glucose Assay Kit-WST (Dojindo, Kumamoto, Japan) following the manufacturer’s instructions. Lactate levels were measured using the Lactate Assay Kit-WST (Dojindo) according to the manufacturer’s instructions. Glucose consumption and lactate production were normalized to cell numbers. The results were presented as fold-change over the respective controls.

### Quantitative Reverse Transcription-PCR (qRT-PCR) Assay

Total RNA was isolated from cells with the TRIzol reagent (Invitrogen) and reverse-transcribed into cDNA using an M-MLV Reverse Transcriptase Kit (Invitrogen). Quantitative real-time PCR was carried out using SYBR Premix Ex Taq II (Takara, Shiga, Japan) in an ABI-7300 Real-Time PCR system (Applied Biosystems, Foster City, CA). The primers were as follows: human *GLUT1*, sense: 5′-GGCCAAGAGTGTGCTAAAGAA-3′, anti-sense: 5′-ACAGCGTTGATGCCAGACAG-3′; human *MMP-1*, sense: 5′-AAAATTACACGCCAGATTTGCC-3′, anti-sense: 5′-GGTGTGACATTACTCCAGAGTTG-3′; human *MMP-14*, sense: 5′-GGCTACAGCAATATGGCTACC-3′, anti-sense: 5′-GATGGCCGCTGAGAGTGAC-3′; human *Cyclin D1*, sense: 5′-GCTGCGAAGTGGAAACCATC-3′, anti-sense: 5′-CCTCCTTCTGCACACATTTGAA-3′; human TMPO-AS1, sense: 5′-GTGCTGCAGGACCGAGG-3′, anti-sense: 5′-GCTTTGTGTCCGCGAGTTTT-3′, and human *β-actin*, sense: 5′-CATGTACGTTGCTATCCAGGC-3′, anti-sense: 5′-CTCCTTAATGTCACGCACGAT-3′. Gene expression was normalized to *beta-actin* mRNA. The expression of miR-140 and miR-143 was measured using the NCode SYBR GreenER miRNA qRT-PCR analysis kit (Invitrogen). The forward primers for miRNA analysis had the same sequences as the mature miRNAs. The forward primer for U6 was 5′-CGCAAGGATGACACGCAAATTCG-3′. The reverse primer was the NCode miRNA universal qPCR primer (Invitrogen). The expression of miRNAs was calculated relative to U6.

### Subcellular Fractionation Assay

Cytoplasmic and nuclear RNA was extracted using the PARIS Kit (Thermo Fisher Scientific, Carlsbad, CA) according to the manufacturer’s instructions. The qRT-PCR analysis was used to measure the expression ratio of TMPO-AS1 between the cytoplasmic and nuclear fractions. U6 served as the nuclear control, and *GAPDH* was used as the cytoplasmic control. The primers were as follows: human U6, sense: 5′-CTCGCTTCGGCAGCACA-3′; anti-sense: 5′-AACGCTTCACGAATTTGCGT-3′ and human *GAPDH* (sense: 5′-ACAACTTTGGTATCGTGGAAGG-3′; anti-sense: 5′-GCCATCACGCCACAGTTTC-3′).

### Luciferase Reporter Assay

The wild-type (WT) human TMPO-AS1 fragment containing the predicted miRNA targeting sites was amplified and cloned into the pGL3-basic vector (Promega, Madison, WI). A human *GLUT1* 3′-untranslated region (3′-UTR) luciferase reporter vector was purchased from OriGene (Rockville, MD). The mutated (MUT) miRNA binding sites in TMPO-AS1 and the *GLUT1* 3′-UTR were generated using a QuickChange site-directed mutagenesis kit (Stratagene, La Jolla, CA). EC cells were co-transfected with the WT or MUT TMPO-AS1 or *GLUT1* 3′-UTR luciferase reporter vector, miR-140/miR-143 mimic, miR-140/miR-143 inhibitor (or the respective control), and renilla luciferase plasmid pRL-CMV (Promega, Madison, WI) using the Lipofectamine 3000 reagent (Invitrogen). The activities of firefly and renilla luciferase in each group were measured 48 hours after transfection using a dual-luciferase reporter assay system (Promega). The firefly luciferase activities were normalized to renilla luciferase activities.

### RNA Immunoprecipitation (RIP) Assay

The RIP assay was conducted using a Magna RIP RNA-Binding Protein Immunoprecipitation Kit (Millipore, Billerica, MA). Briefly, EC cells were re-suspended in RIP lysis buffer. Magnetic beads carrying protein A/G were incubated with anti-Ago2 antibody (Millipore) or control anti-IgG antibody (Millipore) for 30 minutes at room temperature. Lysates were incubated with the bead-antibody complexes in the RIP immunoprecipitation buffer at 4°C overnight. The next day, the RNA-protein-bead complexes were washed in the RIP wash buffer and incubated with proteinase K. After that, the immunoprecipitated RNAs were extracted and subjected to qRT-PCR analysis.

### Statistical Analysis

The results are expressed as the mean ± SD of three independent experiments. Statistical analysis was performed using SPSS 22.0 statistical software (SPSS, Chicago). Differences between the two groups were analyzed using the two-tailed Student’s *t*-tests and Mann-Whitney *U* tests. Comparisons among multiple groups were analyzed using a one-way ANOVA test. A *P* value < 0.05 was considered statistically significant.

## Results

### The Clinical Importance of GLUT1 Expression in EC

**Figure 1:**
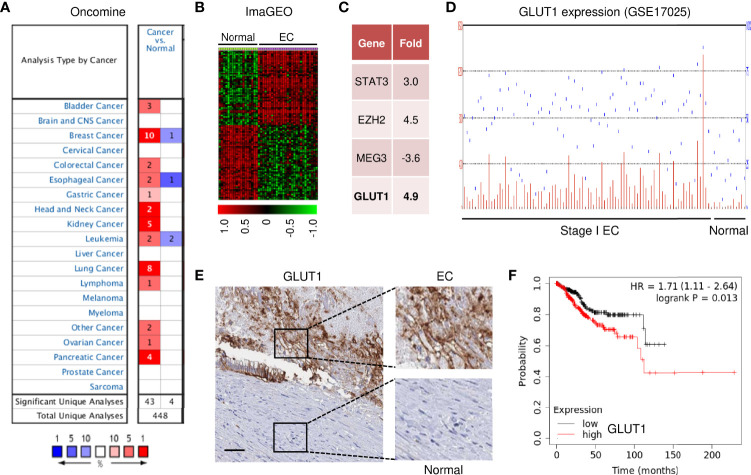
GLUT1 Expression and Clinical Importance in EC. **(A)** The mRNA expression of *GLUT1* (cancer versus normal tissues) in pan-cancers was analyzed with the Oncomine database. The graphic demonstrates the number of datasets that meet our threshold for each cancer type. Red: up-regulation; blue: down-regulation. **(B)** The heatmap was generated by the ImaGEO database using two EC data sets (GSE17025 and GSE56026). **(C)** Up- or down-regulated genes in EC tissues compared to normal tissues (ImaGEO). **(D)** The GEO dataset was analyzed for *GLUT1* expression in stage I EC samples and normal endometrium samples. **(E)** The expression of GLUT1 protein was examined in EC tissue and adjacent normal tissues (Human Protein Atlas database; scale bar: 100 μm). **(F)** The probability of overall survival in EC patients expressing high or low *GLUT1* levels was assessed using the KM plotter database.

**Figure 2:**
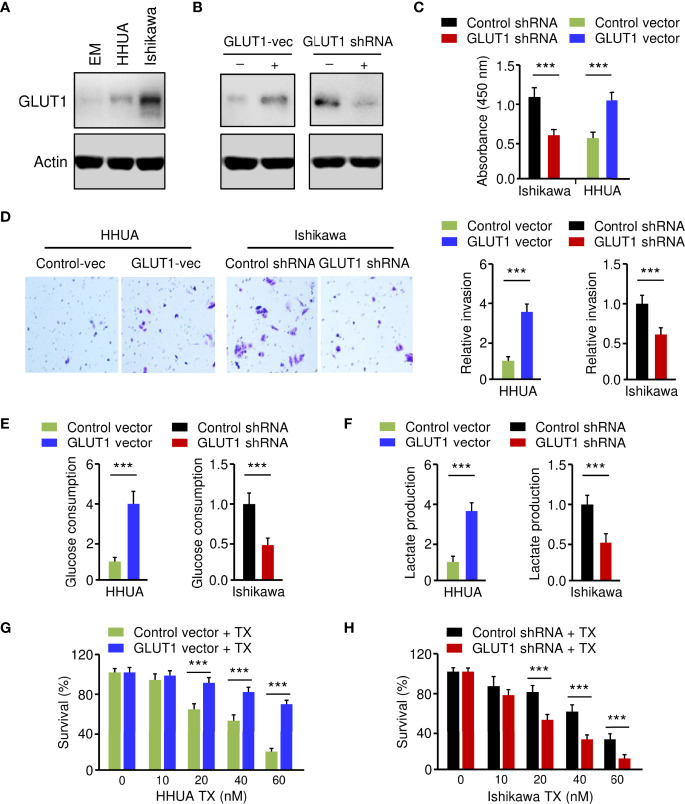
Critical Roles of GLUT1 in Promoting EC Cell Phenotypes. **(A)** Western blotting analysis of GLUT1 protein expression in a normal endometrial cell line (EM) and human EC cell lines. **(B)** Western blotting analysis of GLUT1 expression in GLUT1-overexpressing HHUA cells and Ishikawa cells with GLUT1 silencing. **(C–F)** EC cell proliferation **(C)**, invasion **(D)**, glucose consumption **(E)**, and lactate production **(F)** assays following GLUT1 overexpression or knockdown. **(G, H)** GLUT1-overexpressing HHUA cells **(G)** and Ishikawa cells after knockdown of GLUT1 **(H)** were treated with different concentrations of paclitaxel, and cell viability was examined using the CCK-8 assay. Vec: vector. ***P < 0.001.

To investigate the expression profiles of GLUT1 in human cancer and normal tissues, we used the Oncomine database to perform a pan-cancer transcriptome analysis on its available data sets. The mRNA differences between cancer and normal samples were analyzed using the default selection criteria. There were 448 Oncomine datasets involving GLUT1 (**Figure 1A**). Up-regulation of GLUT1 was observed in various cancer tissues (including bladder, breast, cervical, liver, ovarian, pancreatic cancer, and EC) (**Figure 1A**). To explore the clinical significance of GLUT1 in EC, we performed a meta-analysis of two GEO EC datasets (GSE17025 and GSE56026) using the ImaGEO database. As a result, we selected the differentially expressed genes in EC compared to normal tissues (**Figure 1B**). Remarkably, EC patients had higher levels of known oncogenes (including *STAT3* and *EZH2*) and lower levels of the known tumor suppressor lncRNA MEG3 when compared to normal samples (**Figure 1C**). This analysis also showed a marked up-regulation of *GLUT1* in ECs (**Figure 1C**). Additionally, microarray gene expression data from the GEO website demonstrated that *GLUT1* was overexpressed in stage I EC tissues compared with normal endometrial tissues (**Figure 1D**). Furthermore, we assessed the expression of GLUT1 protein in an EC tissue microarray by extracting IHC images from the Human Protein Atlas database. GLUT1 exhibited both strong and diffuse positive staining in EC, but not in adjacent normal tissues (**Figure 1E**). Subsequently, we sought to determine the clinical relevance of GLUT1 expression in terms of prognosis in EC patients. Kaplan–Meier analysis using the KM plotter database showed that higher GLUT1 expression was significantly associated with worse overall survival in EC patients (**Figure 1F**). Interestingly, GLUT1 expression was low in non-malignant endometrial epithelial cell lines (EM), whereas GLUT1 expression was much higher in EC cell lines (**Figure 2A**). Together, these results suggest that increased GLUT1 expression is linked to EC tumorigenesis and progression, and its overexpression is correlated with poor clinical outcomes.

### Critical Roles of GLUT1 in Promoting EC Cell Phenotypes

To establish the role of GLUT1 in EC cell phenotypes, we generated the HHUA cell line stably overexpressing GLUT1 (GLUT1-vec) and the control cell line (**Figure 2B**). On the other hand, we knocked down the expression of GLUT1 in Ishikawa cells using shRNA for GLUT1 (**Figure 2B**). We first wanted to investigate how GLUT1 overexpression or silencing affects EC cell proliferation, invasion, glucose metabolism, and chemoresistance. Cell functional assays demonstrated that, when compared with control cells, HHUA GLUT1-vec cells showed a significant increase in cell growth, invasion, glucose consumption, lactate production, and paclitaxel resistance (**Figures 2C–G**). However, silencing of GLUT1 expression resulted in a marked decrease in cell proliferation, invasiveness, and glycolysis, as well as sensitization of EC cells to paclitaxel treatment (**Figures 2C–F, H**). These results suggest that GLUT1 is a key player that induces aggressive properties, glycolysis, and chemoresistance in EC cells.

### Pathway Enrichment Analysis of Genes Related to GLUT1 Expression in EC

**Figure 3:**
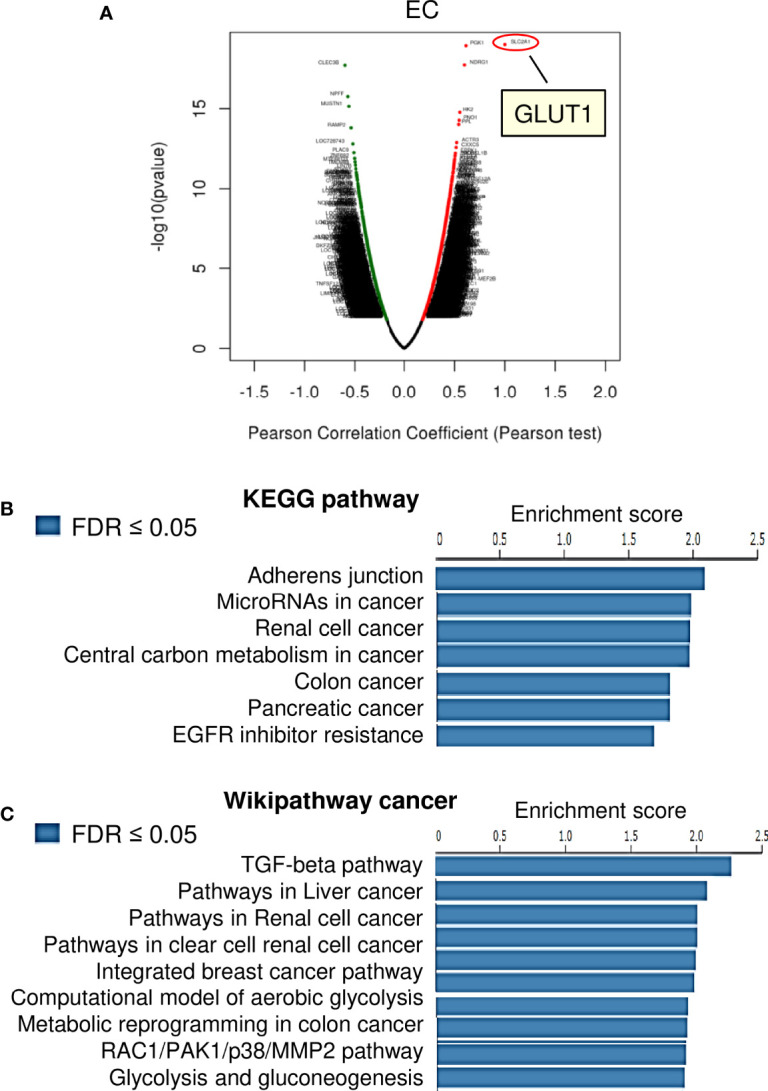
KEGG Pathway and Wiki Pathway Enrichment Analysis of *GLUT1*-Associated Genes in EC. **(A)** Genes that were found to be highly co-expressed with *GLUT1* in the TCGA EC dataset from the LinkedOmics database were chosen. **(B, C)** Gene set enrichment analysis for positively correlated genes of *GLUT1* in TCGA EC tissues. Enrichment analysis for KEGG pathways **(B)** and Wikipathway cancer **(C)** was performed using the LinkedOmics database.

We investigated the genes associated with *GLUT1* in the TCGA EC dataset (n=304), as well as the pathways involved, using the LinkedOmics online database. There were 6648 genes, represented by red dots, which had a positive connection with *GLUT1*. Conversely, there were 13250 genes, represented by green dots, which had a notably negative correlation with *GLUT1* in TCGA EC tissues (**Figure 3A**). Then, we selected the top 100 positively and 100 negatively correlated genes, and performed Kyoto Encyclopedia of Genes and Genomes (KEGG) pathway enrichment analysis. The results of KEGG pathway analysis revealed that the genes that were positively related to *GLUT1* in EC tissues were mainly located in several pathways, including adherens junction, microRNAs in cancer, renal cell cancer, central carbon metabolism in cancer, colon cancer, pancreatic cancer, and EGFR inhibitor resistance (**Figure 3B**). Furthermore, using the GSEA tool in LinkedOmics, Wikipathway cancer enrichment of *GLUT1* co-expressed genes was performed. This enrichment analysis showed that those co-expressed genes were involved in the TGF-beta pathway, pathways in liver cancer, pathways in renal cell cancer, pathways in clear cell renal cell cancer, integrated breast cancer pathway, computational model of aerobic glycolysis, metabolic reprogramming in colon cancer, RAC1/PAK1/p38/MMP2 pathway, and glycolysis and gluconeogenesis (**Figure 3C**). These results indicate the broad impact of GLUT1 on the global transcriptome in EC cells, and support the notion that GLUT1 has a critical role in endometrial tumorigenesis, EC progression, and chemoresistance beyond working as a glucose transporter.

### GLUT1 Is a Positive Upstream Regulator of MMP1, MMP14, and Cyclin D1

**Figure 4:**
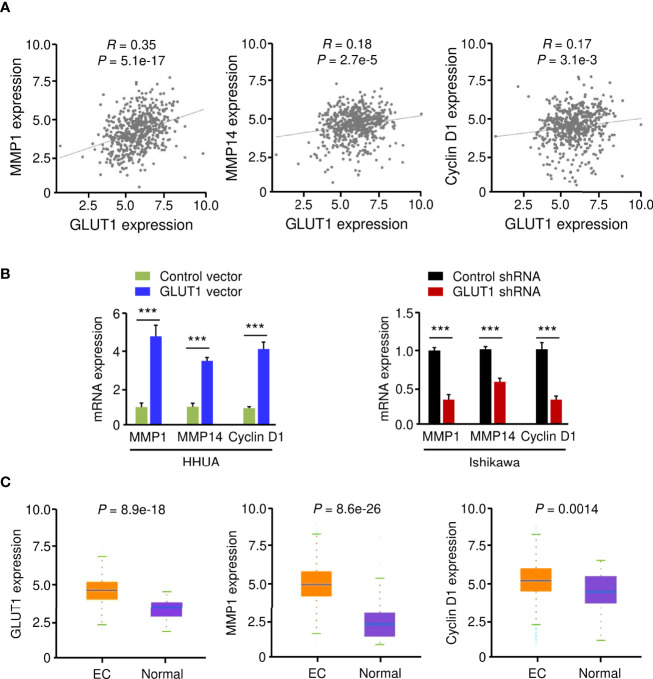
GLUT1 is a Positive Upstream Regulator of MMP1, MMP14, and Cyclin D1. **(A)** The expression of *GLUT1* was positively correlated with the expression of *MMP1, MMP14*, and *Cyclin D1* in EC tissues from the TCGA dataset. **(B)** qRT-PCR analysis of the indicated genes in EC cells after overexpression or knockdown of GLUT1. **(C)** The expression of *MMP1, MMP14*, and *Cyclin D1* in TCGA EC and normal tissues (ENCORI database). ***P < 0.001.

Using the KEGG enrichment analysis, based on the LinkedOmics database, we determined that several cancer-linked pathways (including MicroRNAs in Cancer Pathway) were among the top ten pathways that were significantly related to GLUT1 expression in TCGA EC tissues (**Figure 3B**). Importantly, Cyclin D1 is a key component of the MicroRNAs in the Cancer Pathway (**Figure 3B**), and it controls chemoresistance and glycolysis in human tumor cells (20, 21). In addition, MMP1, MMP14, and Cyclin D1 have important roles in increasing EC cell proliferation, migration, and invasion (22–25). Therefore, we studied whether the levels of MMP1/MMP14/Cyclin D1 were correlated with GLUT1 expression in ECs. By assessing EC samples in TCGA through the ENCORI database, we found that the expression of *GLUT1* was positively correlated with the expression of *MMP1*, *MMP14*, and *Cyclin D1* (**Figure 4A**), indicating that the mechanisms underlying the action of GLUT1 may be related to these genes.

To test this hypothesis, we evaluated the mRNA expression of *MMP1*, *MMP14*, and *Cyclin D1* in EC cells following overexpression or knockdown of GLUT1. By qRT-PCR assay, we found that the levels of *MMP1*, *MMP14*, and *Cyclin D1* were significantly higher in GLUT1-overexpressing cells than in control cells (**Figure 4B**). Additional qRT-PCR analysis also showed that GLUT1 silencing significantly reduced the expression of *MMP1*, *MMP14*, and *Cyclin D1* (**Figure 4B**). We further analyzed the expression of these genes in TCGA EC and normal samples using the ENCORI database. *MMP1*, *MMP14*, and *Cyclin D1* were significantly higher in EC tissues than in normal tissues (**Figure 4C**). Together, these data suggest that MMP1, MMP14, and Cyclin D1 are critical downstream targets of GLUT1 in EC cells.

### GLUT1 Is a Target Gene of MiR-140 and MiR-143

**Figure 5:**
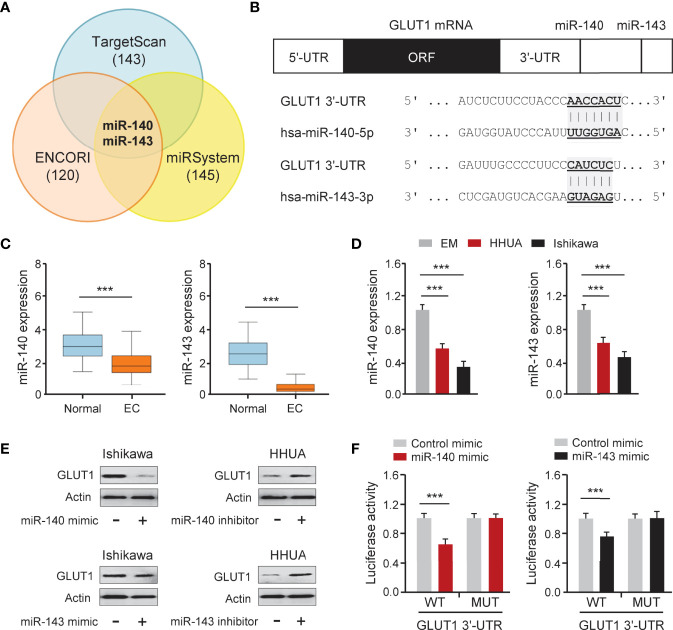
GLUT1 is a Target Gene of MiR-140 and MiR-143. **(A)** Venn diagram showing the overlapping miRNAs predicted by three online databases. **(B)** Computational prediction of duplex formation between miR-140/miR-143 and the *GLUT1* 3′-UTR region. **(C)** The expression of miR-140/miR-143 in TCGA EC tissues and normal tissues (miR-TV database). **(D)** qRT-PCR analysis of miR-140/miR-143 in EC and EM cells. **(E)** GLUT1 protein expression was examined using western blotting assays in EC cells transfected as indicated. **(F)** Luciferase reporter assays in Ishikawa cells transfected with the wild-type (WT) or mutant (MUT) *GLUT1* 3′-UTR, as well as miR-140/miR-143 mimic or a control mimic. ***P < 0.001.

To determine whether overexpression of GLUT1 was caused by miRNA suppression, we searched miRNAs that might bind to the 3′-UTR of *GLUT1* mRNA using three online miRNA target prediction databases (TargetScan, ENCORI, and miRSystem) (**Figure 5A**). Six miRNAs were found in all three databases. Analysis of TCGA EC data from the miR-TV database (http://mirtv.ibms.sinica.edu.tw/) suggested that, among these 6 candidates, only miR-140 and miR-143 were significantly down-regulated in EC tissues compared to normal tissues (**Figures 5B, C**). Our qRT-PCR assay confirmed that EC cell lines had lower expression of miR-140 and miR-143 than EM cells (**Figure 5D**). The above results indicated that the dysregulation of miR-140 and miR-143 may be involved in GLUT1 overexpression in ECs. Thus, we tested the effects of miR-140 and miR-143 on GLUT1 protein expression in EC cells. The results from western blotting analysis showed that overexpression of miR-140 and miR-143 decreased, while inhibition of miR-140 and miR-143 increased the protein expression of GLUT1 in EC cells (**Figure 5E** and **Supplementary Figure 1**). To explore whether miR-140 and miR-143 directly bind to the 3′-UTR region of *GLUT1* mRNA, we performed a dual-luciferase reporter assay. The results showed that the introduction of miR-140 and miR-143 significantly decreased the luciferase activities of WT *GLUT1* 3′-UTR in Ishikawa cells, and had no effect on MUT *GLUT1* 3′-UTR (**Figure 5F**), proving that miR-140 and miR-143 directly target GLUT1 in EC cells. Overall, we confirmed that GLUT1 is a direct target of miR-140/miR-143, and that loss of miR-140/miR-143 expression contributes to GLUT1 up-regulation in ECs.

### MiR-140 and MiR-143 Inhibit EC Cell Glycolysis and Chemotherapeutic Resistance

**Figure 6:**
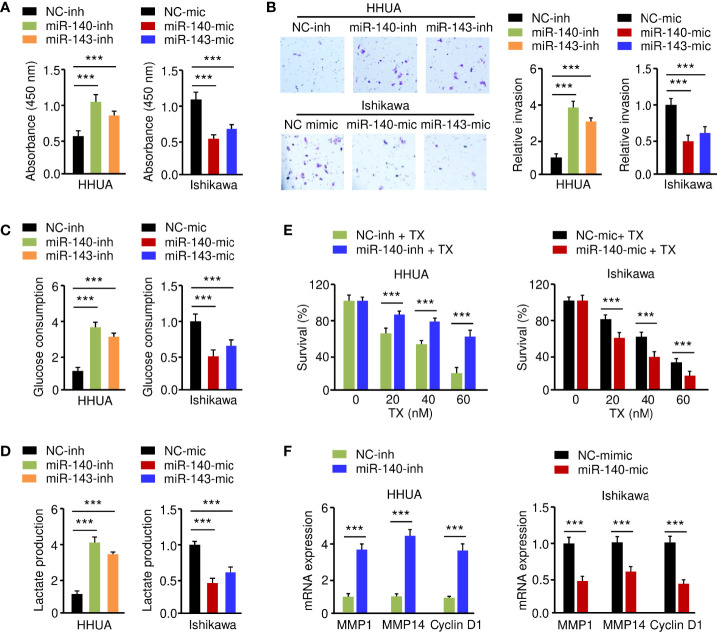
MiR-140 and miR-143 Suppress Glycolysis and Chemoresistance of EC Cells. **(A–D)** Proliferation **(A)**, invasion **(B)**, glucose consumption **(C)**, and lactate production **(D)** assays in EC cells after miR-140 or miR-143 overexpression or knockdown. **(E)** HHUA cells transfected with miR-140 inhibitor (inh) and Ishikawa cells transfected with miR-140 mimic (mic) were treated with different concentrations of paclitaxel, and cell viability was examined using the CCK-8 assay. **(F)** The mRNA expression of the indicated genes was examined in Ishikawa cells after overexpression of miR-140, and in HHUA cells after the knockdown of miR-140. ***P < 0.001.

To elucidate the functional effect of miR-140 and miR-143 on EC cell phenotypes, we increased the expression of miR-140 and miR-143 in Ishikawa cells, while suppressing their expression in HHUA cells. The results from functional assays demonstrated that up-regulation of both miR-140 and miR-143 significantly inhibited the growth, invasion, glucose consumption, and lactate production of Ishikawa cells, whereas knockdown of miR-140 and miR-143 significantly enhanced these malignant phenotypes in HHUA cells (**Figures 6A–D**). We detected the impact of miR-140 and miR-143 expression on paclitaxel treatment and found that overexpression of miR-140 and miR-143 improved the sensitivity of EC cells to paclitaxel (**Figure 6E**), whereas suppression of miR-140 and miR-143 expression inhibited EC cell sensitivity (**Supplementary Figure 2A**). Since *MMP1*, *MMP14*, and *Cyclin D1* are downstream targets of GLUT1, we wanted to investigate whether miR-140 and miR-143 had any effect on their expression. Utilizing a qRT-PCR assay, we demonstrated that the expression of *MMP1*, *MMP14*, and *Cyclin D1* was increased in miR-140/miR-143-silenced HHUA cells, but decreased in Ishikawa cells overexpressing miR-140/miR-143 (**Figure 6F**; **Supplementary Figure 2B**). These results support the idea that miR-140 and miR-143 suppress EC cell proliferation, invasion, and glycolysis, and enhance the chemosensitivity of EC cells to paclitaxel by down-regulating GLUT1.

### LncRNA TMPO-AS1 Interacts With MiR-140/MiR-143 to Inhibit Their Expression

**Figure 7:**
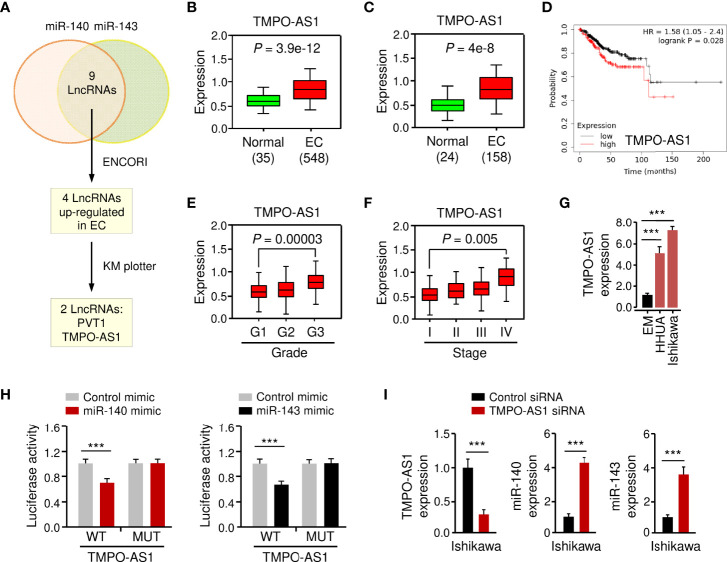
LncRNA TMPO-AS1 Interacts with MiR-140 and MiR-143 to Inhibit Their Expression. **(A)** Workflow for identifying possible lncRNAs that regulate the expression of miR-140 and miR-143 simultaneously. **(B, C)** Expression of TMPO-AS1 in TCGA EC and normal tissues (**B**: ENCORI database; **C**: Wanderer database). **(D)** The probability of overall survival in EC patients expressing high or low TMPO-AS1 levels was examined using the KM plotter database. **(E, F)** TMPO-AS1 expression analysis in EC samples with varying histologic grades (**E**: well (G1), moderate (G2), and poor (G3)) and tumor stages (**F**: I–IV) (TANRIC database). **(G)** qRT-PCR analysis of TMPO-AS1 expression in EC and EM cells. **(H)** Luciferase reporter assays in Ishikawa cells transfected with wild-type (WT) or mutant (MUT) TMPO-AS1 fragments, as well as miR-140/miR-143 mimic or control mimic. **(I)** The expression of TMPO-AS1, miR-140, and miR-143 in Ishikawa cells transfected with TMPO-AS1 siRNA (or control siRNA) was examined using qRT-PCR analysis. ***P < 0.001.

LncRNAs have been shown to interact with miRNAs to regulate the expression of downstream genes (26, 27). To identify lncRNAs that might compete for the binding sites of miR-140/miR-143 with *GLUT1* mRNA, the ENCORI database was used for target prediction. We detected the binding sites for lncRNAs and miR-140/miR-143. In total, 9 lncRNAs may simultaneously bind to miR-140 and miR-143 (**Figure 7A**; **Supplementary Figure 4A**). According to the analysis of TCGA EC data ***via*** the ENCORI database and the Wanderer database, we found that 4 lncRNAs were highly expressed in EC tissues (**Figures 7A–C**). The data from the KM plotter database indicated that PVT1 and TMPO-AS1 (but not the other two lncRNAs) were poor prognostic factors in EC patients (**Figure 7D**) and caught our attention. Because PVT1 functions as an oncogenic molecule in various cancers (28), TMPO-AS1 was chosen as the miR-140/miR-143-associated lncRNA in our study.

Basic information about TMPO-AS1 was obtained from the Lnc2Catlas database (https://lnc2catlas.bioinfotech.org). TMPO-AS1 is an intergenic lncRNA localized in chr12:98512973–988516422 and annotated as ENSG00000257167.2 in the ENSEMBL database (**Supplementary Figure 3A**). The lncATLAS database (https://lncatlas.crg.eu/) was applied to determine the subcellular localization of TMPO-AS1 in human cells. The results showed that TMPO-AS1 was mainly found in the cytoplasm of human cancer cells (including A549, HT1080, and MCF-7 cells) (**Supplementary Figure 3B**). Furthermore, qRT-PCR analysis of EC cell nuclear and cytoplasmic fractions revealed that TMPO-AS1 is mainly located in the cytoplasm (**Supplementary Figure 3C**).

To further explore whether TMPO-AS1 expression levels correlate with the clinicopathological characteristics of EC, we analyzed the expression of TMPO-AS1 in TCGA EC patients using the TANRIC database. We noticed that TMPO-AS1 expression was significantly increased in higher grade (grade 3) ECs than in lower grade (grade 1) ECs (**Figure 7E**). Furthermore, TMPO-AS1 levels were significantly increased in stage IV ECs compared to stage I ECs (**Figure 7F**). As expected, TMPO-AS1 expression was induced in EC cell lines when compared to normal EM cells (**Figure 7G**).

To confirm the relationship between TMPO-AS1 and miR-140/miR-143, we applied a luciferase assay. Transfection with miR-140 or miR-143 mimic markedly inhibited the WT TMPO-AS1-driven luciferase activities, but did not affect the MUT TMPO-AS1-driven luciferase activities (**Figure 7H**; **Supplementary Figure 4A**). Consistently, TMPO-AS1 negatively suppressed the expression of miR-140 and miR-143 in EC cells (**Figure 7I**; **Supplementary Figure 4B**). To further verify the binding of TMPO-AS1 and miR-140/miR-143, we performed an RIP assay. Endogenous TMPO-AS1 was significantly enriched in miR-140/miR-143 mimic-transfected EC cells compared with control cells transfected with control mimic (**Supplementary Figure 4C**). These results suggest that TMPO-AS1 negatively regulates the expression of miR-140 and miR-143 through direct interactions.

### TMPO-AS1 Promotes GLUT1 Expression by Suppressing MiR-140 and MiR-143 Levels

**Figure 8:**
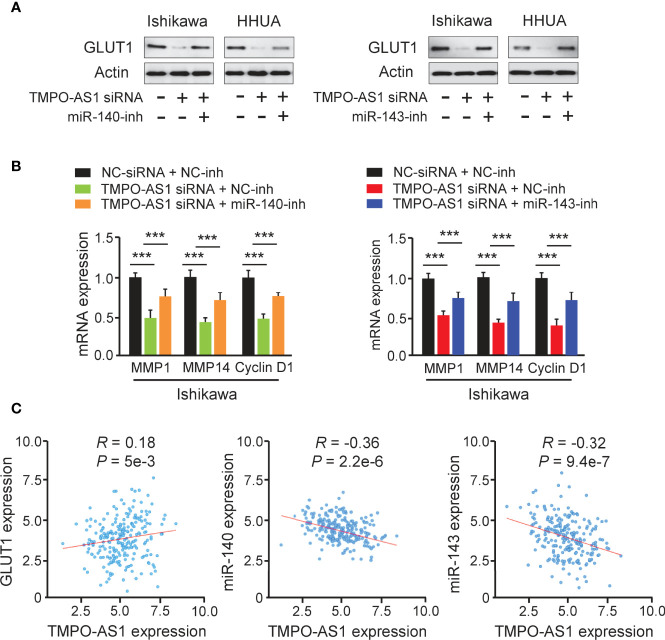
TMPO-AS1 Induces GLUT1 Expression by Repressing MiR-140 and MiR-143 Levels. **(A)** GLUT1 protein expression was measured in Ishikawa and HHUA cells transfected with (or without) TMPO-AS1 siRNA, along with (or without) miR-140/miR-143 inhibitor. **(B)** Examination of *MMP1*, *MMP14*, and *Cyclin D1* expression in Ishikawa cells transfected as indicated. **(C)** Correlation analysis between TMPO-AS1 and GLUT1 or between TMPO-AS1 and miR-140/miR-143. ***P < 0.001.

Because TMPO-AS1 functions as a competing endogenous RNA for miR-140 and miR-143, we wondered whether TMPO-AS1 could modulate the expression of GLUT1 by negatively regulating these two miRNAs. To test this possibility, we transfected Ishikawa and HHUA cells with TMPO-AS1 siRNA or control siRNA, along with (or without) miR-140/miR-143 inhibitor, and measured the protein expression of GLUT1 using western blotting analysis. Knockdown of TMPO-AS1 decreased the expression of GLUT1, but inhibition of miR-140 and miR-143 restored GLUT1 expression in Ishikawa and HHUA cells (**Figure 8A**). qRT-PCR assays were used to estimate the impact of TMPO-AS1 silencing, in the presence (or absence) of the miR-140/miR-143 inhibitor, on *MMP1*, *MMP14*, and *Cyclin D1* expression. The mRNA levels of *MMP1*, *MMP14*, and *Cyclin D1* in EC cells were decreased greatly with the knockdown of TMPO-AS1 and increased when miR-140 and miR-143 were inhibited (**Figure 8B**). To investigate the clinical correlation between TMPO-AS1 and GLUT1 levels in EC patient tissues, we obtained the expression data of 548 EC samples from the TCGA project in the ENCORI database. Then, we calculated the Pearson correlation for TMPO-AS1 and *GLUT1* and found a positive and significant correlation between them (**Figure 8C**). Conversely, the levels of miR-140 and miR-143 were negatively correlated with TMPO-AS1 expression in our analysis (**Figure 8C**). These correlations in the TCGA EC dataset were highly consistent with our above results (**Figures 5**, **7**). Together, TMPO-AS1 promotes GLUT1 expression by negatively regulating miR-140 and miR-143.

### Knockdown of TMPO-AS1 Inhibits Glycolysis and Reverses Chemoresistance of EC Cells

**Figure 9:**
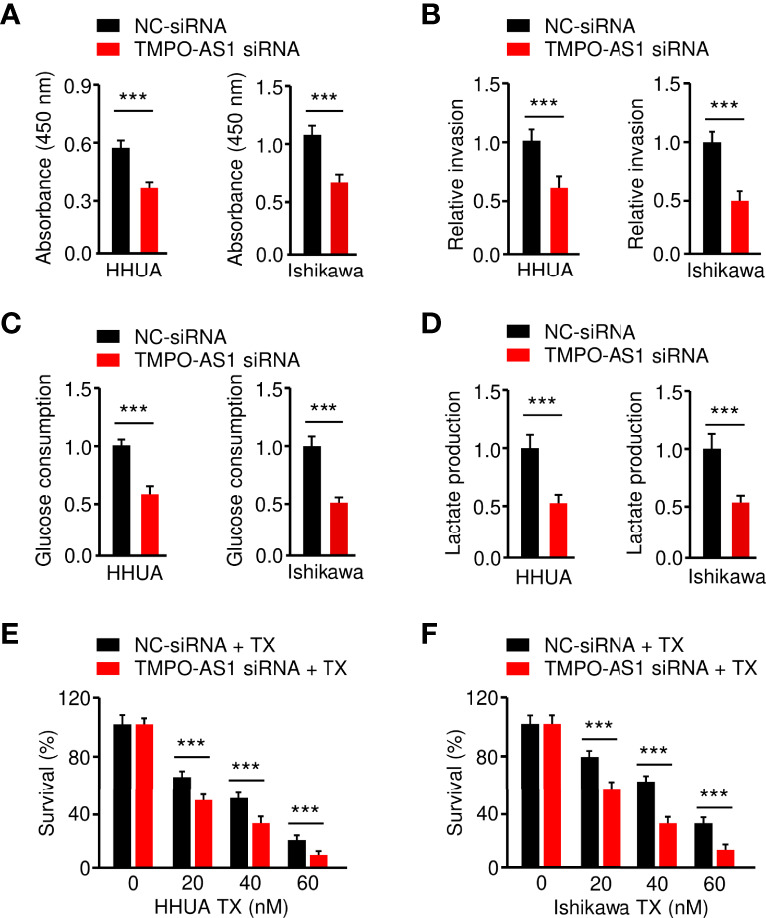
Knockdown of TMPO-AS1 Inhibits Glycolysis and Reverses Chemoresistance of EC Cells.**(A–D)** *Proliferation* **(A)**, invasion **(B)**, glucose consumption **(C)**, and lactate production **(D)** assays in EC cells after the knockdown of TMPO-AS1. **(E, F)** HHUA **(E)** or Ishikawa cells **(F)** were transfected with TMPO-AS1 siRNA (or control siRNA) and treated with different concentrations of paclitaxel. Cell viability was examined using a CCK-8 assay. ***P < 0.001.

Finally, we investigated whether suppressing TMPO-AS1 expression is effective in inhibiting EC cell growth, invasion, glycolysis, and chemoresistance. CCK-8, invasion, glucose consumption, lactate production, and drug sensitivity assays demonstrated that siRNA-mediated TMPO-AS1 downregulation significantly reduced EC cell growth, invasion, glucose consumption, and lactate production, as well as reversing EC cell resistance to paclitaxel (**Figures 9A–F**). These results link the up-regulation of TMPO-AS1 to increased glycolytic metabolism and the development of paclitaxel resistance, suggesting potential therapeutic strategies that target TMPO-AS1 to improve the treatment of paclitaxel-resistant EC.

## Discussion

With over 319,000 cases diagnosed worldwide, EC is the most common gynecologic cancer in developed countries (29). Early cancer detection is crucial for improving patient survival rates by providing care at the earliest possible stage. Although blood- and tissue-based biomarker candidates for EC detection have been reported, to date, there are no validated biological markers that can reliably detect EC at an early stage (30). The GLUT1 protein is rarely expressed in normal endometrium, but was abundant in pre-cancerous endometrial lesions and most EC tissues (13, 14). Here, we have shown that GLUT1 is exclusively expressed in stage I EC samples and not in normal endometrium, and high GLUT1 expression is closely related to unfavorable outcomes. These findings imply that GLUT1 could be used as a diagnostic and prognostic marker for EC. More importantly, GLUT1 expression is elevated across a wide range of cancer types, implying that GLUT1 could be used to screen for other cancers.

Several oncogenes and tumor suppressor genes, including p53 (31), HER2 (32), PI3KCA (32), and PTEN (32), have previously been identified as regulators in EC. However, most of these could be used to predict prognosis, and valuable therapeutic targets are still lacking in EC. In this study, we discovered that GLUT1 is required for EC cell proliferation, invasion, glucose consumption, and lactate production. Furthermore, shRNA-induced repression of GLUT1 results in a remarkable reduction in these malignant properties of EC cells, substantiating that GLUT1 is a potentially targetable biomarker for EC.

To date, many natural and synthetic GLUT1 inhibitors have shown anti-cancer effectiveness (10, 33, 34). A small-molecule inhibitor of GLUT1, WZB117, was shown to induce cell death in lung and breast cancer cells while having no effect on normal cells (35). Recently, BAY-876, a highly selective GLUT1 inhibitor, was recently reported to inhibit the tumorigenesis of ovarian cancer cells *in vivo* by blocking glycolysis and growth (36). Notably, treatment with BAY-876 suppresses cell viability and glucose uptake in spheroid cells derived from EC patient tissues (16), indicating the possibility that the blockade of GLUT1 might inhibit the characteristics of EC stem cells. In line with these results, we verified that inhibiting GLUT1 expression with shRNA could effectively sensitize the representative EC cell line Ishikawa to paclitaxel *in vitro*. Thus, inhibiting GLUT1 expression and its function might be explored as a potential therapeutic target to delay EC progression and combat chemoresistance in this cancer.

Aside from its well-characterized role in tumor glycolysis, GLUT1 exhibits diverse functions in different aspects of tumorigenesis and metastasis, including proliferation, motility, invasiveness, and epithelial-mesenchymal transition (6, 7, 10, 37). GLUT1 acts on tumor cells *via* a number of downstream signaling pathways, including the EGFR/ERK/PKM2 and integrin 1/Src/FAK signaling pathways (6, 7). In addition, GLUT1 has been shown to regulate many proteins, including Cyclin D1 (6, 7), MMP1 (37), MMP2 (38), and MMP14 (39). Cyclin D1 overexpression confers resistance to cisplatin- or paclitaxel-mediated apoptosis in human cancer cells (40, 41). Both MMP1 (23) and MMP14 (24) are overexpressed in EC and are crucial for EC metastasis. Here, we found that up-regulation of GLUT1 significantly increases the expression of *MMP1*, *MMP14*, and *Cyclin D1* in EC cells, implying that GLUT1 promotes EC cell invasion and paclitaxel resistance, at least in part by increasing MMP1/MMP14/Cyclin D1 expression.

GLUT1 expression is controlled by transcription factors, lncRNAs, and tumor suppressor miRNAs (8). Transcription factors (including hypoxia-inducible factor HIF-1α and c-Myc) bind directly to the promoter region of *GLUT1* and activate its transcription (8). Activation of the RAS pathway and PI3K/AKT pathway is also implicated in the up-regulation of GLUT1 in cancer cells (8). Furthermore, lncRNA HOTAIR is able to up-regulate GLUT1 expression in hepatocellular carcinoma cells (8). Moreover, multiple miRNAs (such as miR-140 and miR-143) have been shown to directly regulate GLUT1 expression in breast cancer (42) and T cells (43), respectively. Interestingly, the aberrant expression of both miR-140 and miR-143 has been detected in various cancers, including EC (44, 45). MiR-143 is recognized as a powerful tumor suppressor miRNA that inhibits cancer progression, glycolysis, and chemoresistance by regulating a large number of oncogenes (44). MiR-140 is expressed at low levels in EC tissues (46, 47), but its cellular functions and its target genes remain poorly understood. Here, our study provided the first evidence showing that both miR-140 and miR-143 suppress the growth, invasion, glycolysis, and chemoresistance of EC cells by directly targeting GLUT1. Collectively, post-transcriptional modifications mediated by miR-140/miR-143 could influence GLUT1 expression levels in EC cells.

Considering the down-regulation of miR-140 and miR-143 in ECs, we investigated the mechanism involved in miR-140 and miR-143 regulation. Recent research has indicated the ability of lncRNAs to sponge miRNAs and modulate their expression (18). Consistent with these reports, we have shown that, by sponging miR-140 and miR-143, lncRNA TMPO-AS1 suppresses their expression to induce the expression of GLUT1. Interestingly, TMPO-AS1 is frequently up-regulated in tumor tissues, and it is functionally required for maintaining cell invasiveness and chemoresistance (48, 49). However, its biological functions in EC, as well as the relationship between TMPO-AS1 and glycolysis, are still unclear. Our results demonstrate for the first time that TMPO-AS1 predicts poor prognosis in EC patients, and has a pivotal oncogenic role in enhancing glycolysis and paclitaxel resistance in EC cells, by weakening the suppressive effects of miR-140/miR-143 on GLUT1. Future strategies targeting TMPO-AS1 and GLUT1 may represent a promising therapeutic option for treating paclitaxel-resistant EC.

There are several limitations to our study. One limitation is that, although aberrant overexpression of GLUT1 and TMPO-AS1 was detected in GEO and TCGA EC samples, future studies using primary EC and normal tissues will be needed to verify the clinical relevance of our findings. In addition, EC can be divided into two categories: type I and type II (32). Type I and type II EC exhibit distinct clinical behaviors and molecular mechanisms (50), and Ishikawa and HHUA cells serve as representative models for type I EC (51). Thus, another limitation of our investigation is that we are unable to determine whether this TMPO-AS1-miR-140/miR-143-GLUT1 pathway is functionally active in type II EC. We should evaluate the biological roles of GLUT1 and TMPO-AS1 in type II EC cells. Finally, mouse models of EC will be used to validate the results from the *in vitro* experiments.

## Conclusion

**Figure 10:**
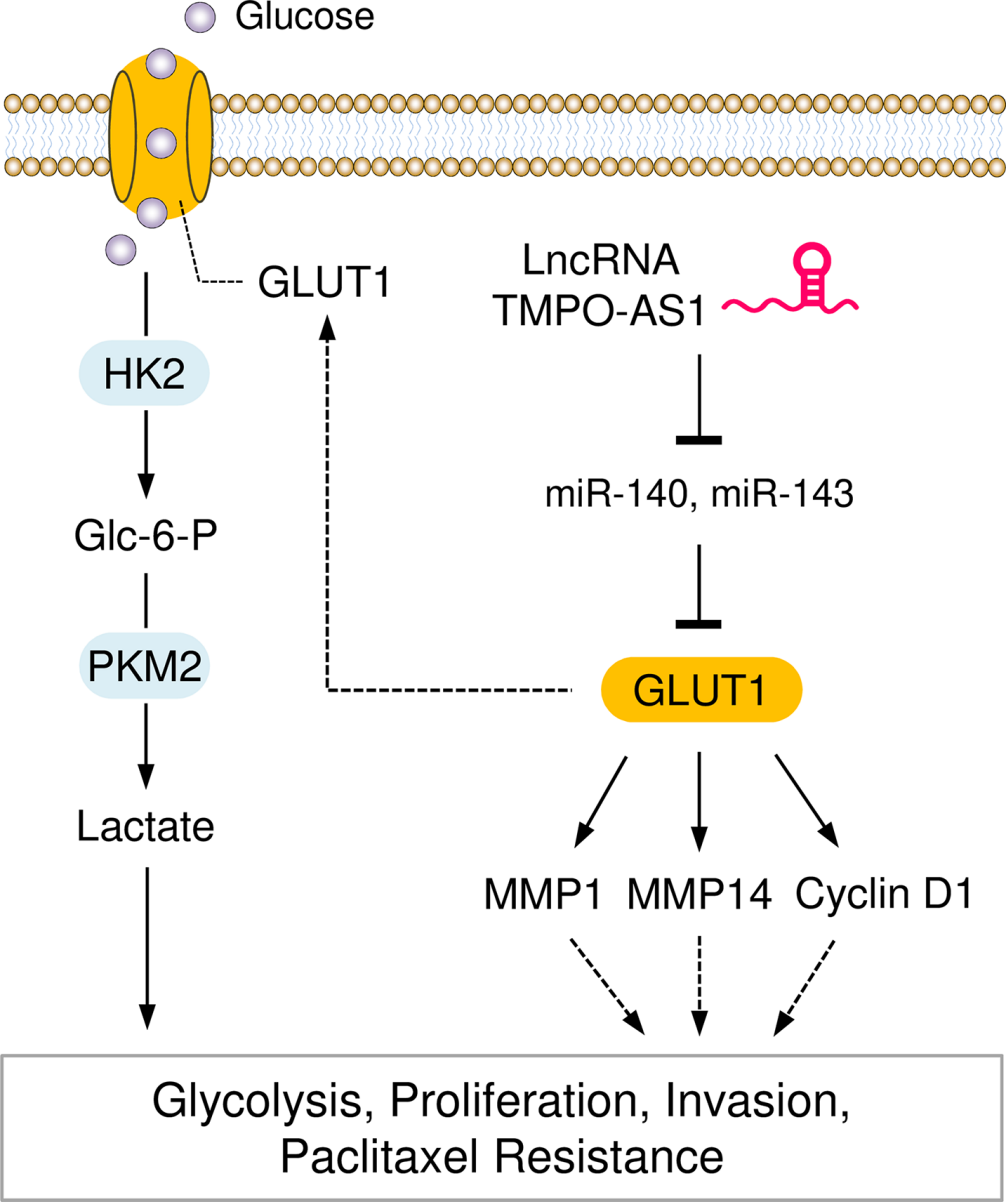
Graphical Abstract showing that the LncRNA TMPO-AS1 Regulates Glycolysis and Paclitaxel Resistance in EC Cells by Affecting the MiR-140/MiR-143-GLUT1 Pathway.

In summary, our study reveals that GLUT1 and lncRNA TMPO-AS1 enhance glycolysis and paclitaxel resistance in EC. TMPO-AS1 acts as a sponge to compete with miR-140 and miR-143, thereby removing the depression of GLUT1 (**Figure 10**). Our findings link the dysregulation of the TMPO-AS1-miR-140/miR-143-GLUT1 pathway to the development of glycolysis and paclitaxel resistance. Therefore, interventions that target this signaling pathway may provide a better chance of slowing EC progression and inhibiting paclitaxel resistance.

## Data Availability Statement

The original contributions presented in the study are included in the article/**Supplementary Material**. Further inquiries can be directed to the corresponding authors.

## Author Contributions

PD, YK, and JY conceived the project. PD, FW, and MT performed the experiments and data collection. YX, YK, KI, NK, and HW analyzed the data. PD wrote the paper. All authors read and approved the final manuscript.

## Funding

This work was supported by a grant from JSPS Grant-in-Aid for Scientific Research (C) (19K09769, 22K09541 and 22K09634), National Natural Science Foundation of China (81873978), Key Project of Social Development in Jiangsu Province (BE2019691), Postdoctoral Research Funding Project of Jiangsu Province (2021K012A), and an NIH/NCI grant 1R21CA216585-01A1 to JY.

## Conflict of Interest

The authors declare that the research was conducted in the absence of any commercial or financial relationships that could be construed as a potential conflict of interest.

## Publisher’s Note

All claims expressed in this article are solely those of the authors and do not necessarily represent those of their affiliated organizations, or those of the publisher, the editors and the reviewers. Any product that may be evaluated in this article, or claim that may be made by its manufacturer, is not guaranteed or endorsed by the publisher.
