# Supplementary material for: Long Non-Coding RNA TMPO-AS1 Promotes GLUT1-Mediated Glycolysis and Paclitaxel Resistance in Endometrial Cancer Cells by Interacting With miR-140 and miR-143
Source: Front Oncol. 2022 May 27;12:912935. doi: 10.3389/fonc.2022.912935 (PMC9195630; doi:10.3389/fonc.2022.912935)
Supplement: Supplementary file 1 [file DataSheet_1.pdf]

Supplementary Material

Long non-coding RNA TMPO-AS1 promotes GLUT1-mediated glycolysis and paclitaxel resistance in endometrial cancer cells by interacting with miR-140 and miR-143

Peixin Dong[1,†,*], Feng Wang[2,†], Mohammad Taheri[3,4,†],Ying Xiong[5], Kei Ihira[1], Noriko Kobayashi[1], Yosuke Konno[1,*], Junming Yue[6,7,*], and Hidemichi Watari[1]

1. Department of Obstetrics and Gynecology, Hokkaido University School of Medicine, Hokkaido University, Sapporo 060-8638, Japan
2. Department of Laboratory Medicine, Affiliated Hospital of Nantong University, Jiangsu, China
3. Skull Base Research Center, Loghman Hakim Hospital, Shahid Beheshti University of Medical Sciences, Tehran, Iran
4. Institute of Human Genetics, Jena University Hospital, Jena, Germany
5. Department of Gynecology, State Key Laboratory of Oncology in South China, Sun Yat-sen University Cancer Center, Guangzhou 510060, China
6. Department of Pathology and Laboratory Medicine, University of Tennessee Health Science Center, Memphis, TN 38163, USA
7. Center for Cancer Research, University of Tennessee Health Science Center, Memphis, TN 38163, USA

† Contributed equally

* Correspondence: Peixin Dong (dpx1cn@gmail.com) or Yosuke Konno (konsuke013@gmail.com) or Junming Yue (jyue@uthsc.edu)

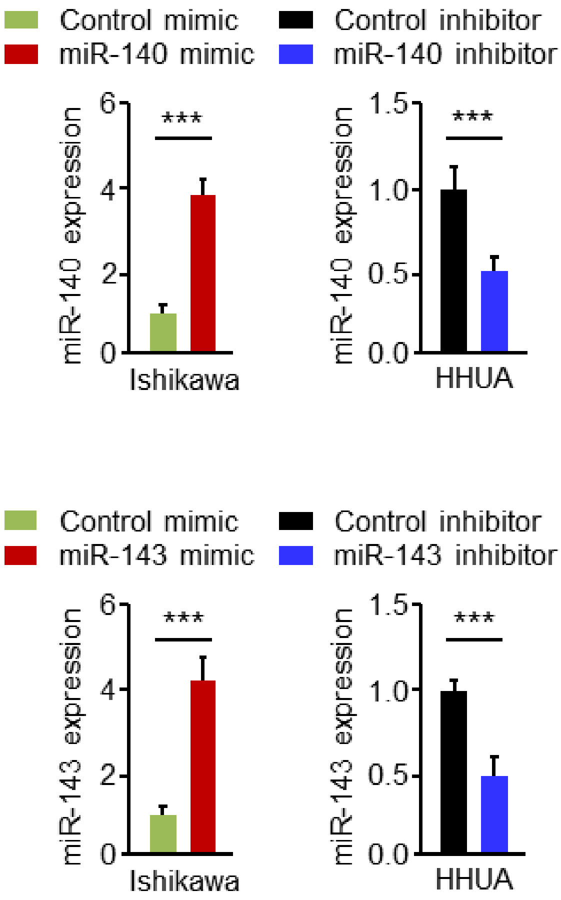

Supplementary Fig. 1: Expression of miR-140 and miR-143 in EC Cells Transfected as Indicated. ****P* < 0.001.

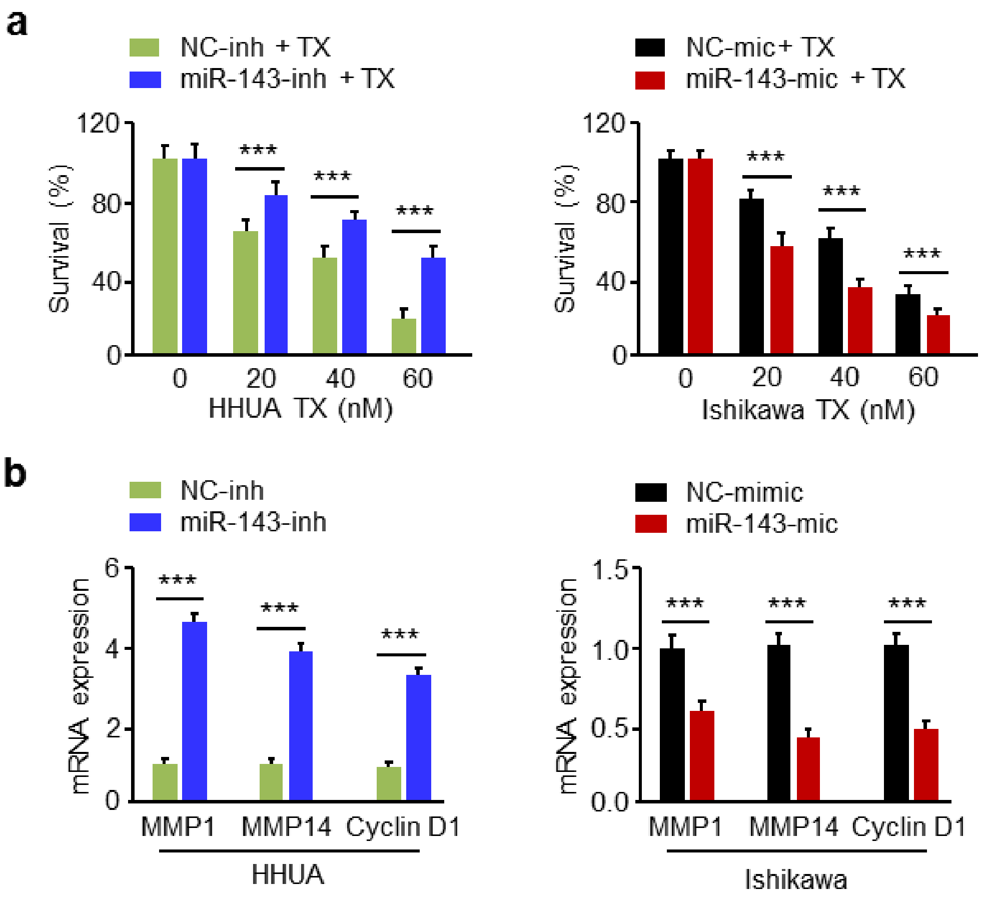

Supplementary Fig. 2: The Effects of MiR-140 and MiR-143 on Paclitaxel Resistance and Gene Expression.

(**A**) HHUA cells transfected with miR-143 inhibitor (inh), and Ishikawa cells transfected with miR-143 mimic (mic) were treated with different concentrations of paclitaxel, and cell viability was examined using CCK-8 assay. (**B**) The mRNA expression of the indicated genes was examined in Ishikawa cells after overexpression of miR-143, and in HHUA cells after knockdown of miR-143. ***$P < 0.001$.

a

TMPO-AS1

Gene ID	ENSG00000257167.2
Location	chr12:98512973-98516422
Strand	-
Class	intergenic
Length	3357
Exons	2
Transcript	ENST00000548760

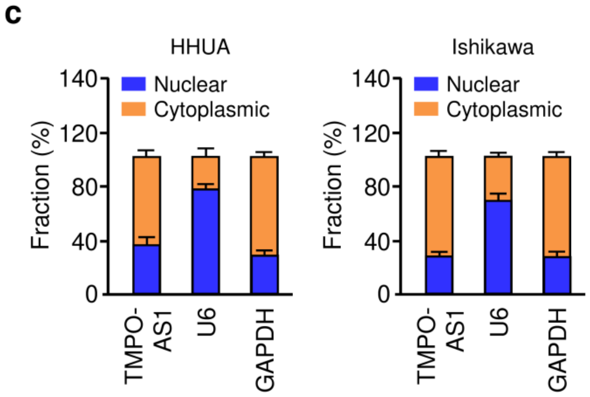

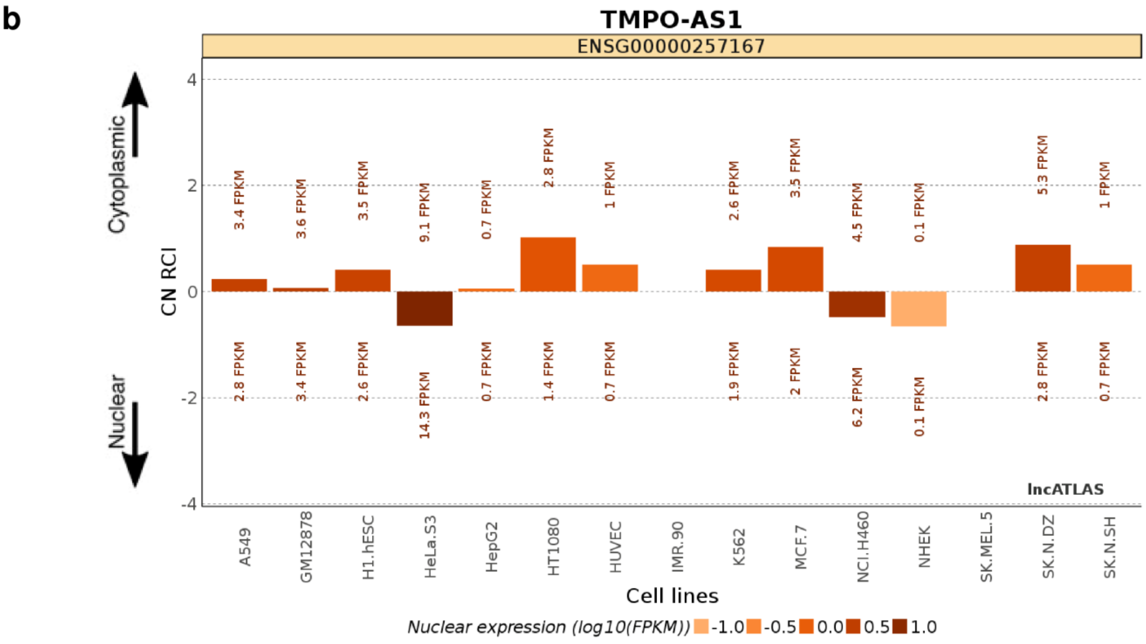

Supplementary Fig. 3: Basic Information of TMPO-AS1.

(**A**) Basic details of TMPO-AS1 were obtained from the Lnc2Catlas database. (**B**) The lncATLAS database was applied to determine the subcellular localization of TMPO-AS1 in human cells. (**C**) The nuclear-cytoplasmic fractionation assay showed that TMPO-AS1 was mainly located in the cytoplasm of EC cells.

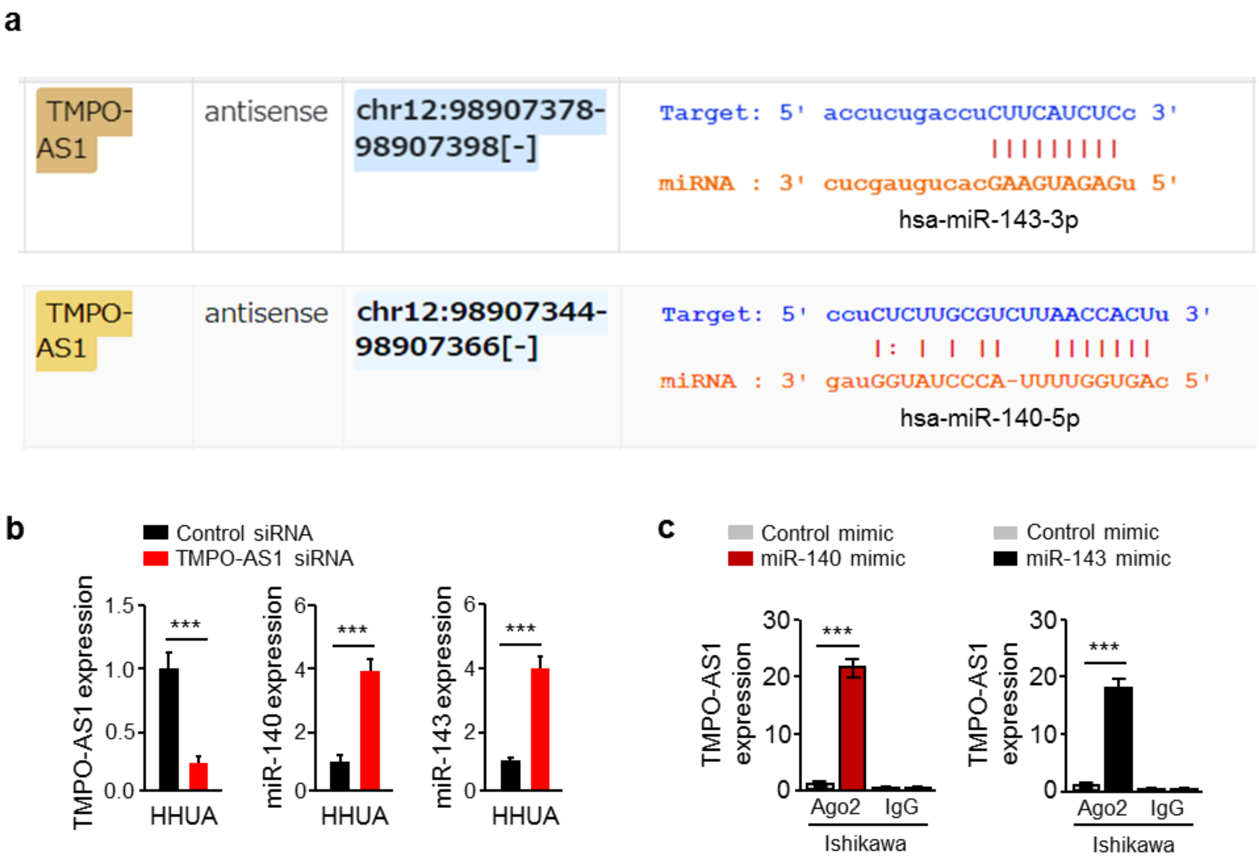

Supplementary Fig. 4: TMPO-AS1 Interacts with MiR-140/MiR-143 and Represses Their Expression in EC Cells.

(**A**) Computational prediction of duplex formation between miR-140/miR-143 with the TMPO-AS1 sequence. (**B**) qRT-PCR analysis of the expression of TMPO-AS1, miR-140 and miR-143 in HHUA cells transfected with TMPO-AS1 siRNA (or control siRNA). (**C**) RIP assay was performed on Ishikawa cells. The levels of TMPO-AS1 were detected by qRT-PCR assay. ***$P < 0.001$.
